# Carbapenem-resistant Enterobacterales and *Pseudomonas aeruginosa* causing infection in Africa and the Middle East: a surveillance study from the ATLAS programme (2018–20)

**DOI:** 10.1093/jacamr/dlac060

**Published:** 2022-06-17

**Authors:** James A Karlowsky, Samuel K Bouchillon, Ramy El Mahdy Kotb, Naglaa Mohamed, Gregory G Stone, Daniel F Sahm

**Affiliations:** IHMA, Schaumburg, IL, USA; Department of Medical Microbiology and Infectious Diseases, Max Rady College of Medicine, University of Manitoba, Winnipeg, Manitoba, Canada; IHMA, Schaumburg, IL, USA; Pfizer Inc., Dubai, UAE; Pfizer Inc., New York, NY, USA; Pfizer Inc., Groton, CT, USA; IHMA, Schaumburg, IL, USA

## Abstract

**Objectives:**

To determine the *in vitro* susceptibility of Enterobacterales (*n = *5457) and *Pseudomonas aeruginosa* (*n = *1949) isolated from hospitalized patients in Africa (three countries) and the Middle East (five countries) in 2018–20 to a panel of 11 antimicrobials and to identify β-lactamase/carbapenemase genes in isolates with meropenem-non-susceptible and/or ceftazidime/avibactam-resistant phenotypes.

**Methods:**

CLSI broth microdilution testing generated MICs that were interpreted using CLSI (2021) breakpoints. β-Lactamase/carbapenemase genes were identified using multiplex PCR assays.

**Results:**

Enterobacterales isolates were highly susceptible to amikacin (96.7%), ceftazidime/avibactam (96.6%) and tigecycline (96.0%), and slightly less susceptible to meropenem (94.3%). In total, 337 Enterobacterales isolates (6.2% of all Enterobacterales isolates) carried one or more carbapenemase genes: 188 isolates carried a serine carbapenemase (178 OXA, 10 KPC) and 167 isolates carried an MBL (18 isolates carried both an MBL and an OXA). NDM-1 was the most common MBL identified (64.1% of NDM enzymes; 59.9% of all MBLs). OXA-48 (47.8%) and OXA-181 (38.8%) were the most common OXAs detected. *P. aeruginosa* isolates were most susceptible to ceftazidime/avibactam (89.1%) and amikacin (88.9%). Only 73.1% of *P. aeruginosa* isolates were meropenem susceptible. The majority (68.1%) of *P. aeruginosa* isolates tested for carbapenemase/β-lactamase genes were negative. In total, 88 isolates (4.5% of all *P. aeruginosa* isolates) carried one or more carbapenemase genes: 81 isolates carried an MBL and 8 carried a GES carbapenemase (1 isolate carried genes for both).

**Conclusions:**

Carbapenemase detection was closely associated with meropenem-non-susceptible phenotypes for Enterobacterales (89.1%) but not for *P. aeruginosa* (24.2%). Wide geographic variation in carbapenemase type and frequency of detection was observed.

## Introduction

Widespread antimicrobial resistance and limited treatment options are increasing challenges for clinicians managing Gram-negative infections in hospital settings around the world. Infections caused by MDR Gram-negative organisms cause significant morbidity and mortality, longer hospitalizations and increased costs compared with infections caused by susceptible organisms.^1^*Pseudomonas aeruginosa* is a leading nosocomial pathogen and causes resistant infections that can be difficult to treat. While the reported extent of coinfection with bacterial pathogens in patients hospitalized with COVID-19 varies, *P. aeruginosa* is among the most frequently identified species in such patients, with a higher proportion in critically ill ICU patients.^2^ Although new compounds have been added to the armamentarium against MDR *P. aeruginosa*, resistance even to these new agents, including the β-lactam/β-lactamase inhibitor combinations, challenges the ability to successfully treat serious infections.

Carbapenem resistance is increasing in clinical isolates of Enterobacterales and *P. aeruginosa* worldwide.^3–8^ WHO classifies carbapenem-resistant and ESBL-producing Enterobacterales and carbapenem-resistant *P. aeruginosa* as critical, priority 1 pathogens^9^ and CDC lists carbapenem-resistant Enterobacterales and MDR (frequently carbapenem-resistant) *P. aeruginosa* as urgent and serious threats, respectively, to national and global health.^10^ The emergence of MDR isolates is a serious public health threat that affects high-risk patients in ICUs, haematology-oncology wards or burn units. Accurate and timely detection of carbapenem-resistant Enterobacterales and *P. aeruginosa* by clinical laboratories, phenotypic and genotypic resistance surveillance, monitoring expansion of mobile genetic resistance elements, antimicrobial stewardship programmes, strict infection prevention and control practice, and the development of new agents and treatment strategies are all important measures to limit the emergence and spread of carbapenem-resistant Gram-negative bacilli and to optimize the treatment of patients infected with these pathogens.

Carbapenemases (serine carbapenemases and MBLs) are pervasive resistance mechanisms in carbapenem-resistant Enterobacterales in some geographic regions with *Klebsiella pneumoniae* being the most common species of Enterobacterales carrying carbapenemases in all locales.^3,4,6,11,12^ Enterobacterales also demonstrate resistance to carbapenems by other mechanisms, typically hyperproduction of AmpC β-lactamases (plasmid or chromosomal), with or without ESBLs (plasmid), in combination with loss of function or reduced expression of outer membrane pore-forming proteins, penicillin-binding protein mutations and/or upregulated efflux.^3,4,11,12^ Global surveillance initiatives have reported wide geographic variation in the prevalence of carbapenem-resistant Enterobacterales and the carbapenemase types carried.^3,4,11–13^ Global surveillance programmes have also shown that carbapenemases are absent in the majority of carbapenem-resistant isolates of *P. aeruginosa*.^4,14^ Carbapenem non-susceptible *P. aeruginosa* commonly demonstrate stable derepression (mutation) of *Pseudomonas*-derived cephalosporinase (PDC), the normally inducible chromosomal AmpC β-lactamase of *P. aeruginosa*, combined with upregulation of efflux pumps (e.g. MexAB-OprM) and/or porin (OprD) loss.^4^

Published data defining resistance mechanisms in carbapenem-resistant Enterobacterales and *P. aeruginosa* isolated from patients in specific countries in Africa and the Middle East are infrequent compared with the availability of data describing isolates from countries in Europe, Asia-Pacific, North America and the Latin and South America regions.^5,13,15–17^ The intent of the current study was to supplement the paucity of available data on resistance patterns and mechanisms by determining the *in vitro* susceptibility of Enterobacterales and *P. aeruginosa* isolated from hospital patients in Africa and the Middle East in 2018–20 to a panel of 11 antimicrobial agents and by identifying β-lactamase/carbapenemase genes in a subset of isolates with meropenem-non-susceptible or ceftazidime/avibactam-resistant phenotypes.

## Materials and methods

### Bacterial isolates and antimicrobial susceptibility testing

The Antimicrobial Testing Leadership and Surveillance (ATLAS) global surveillance programme collected clinical isolates of Enterobacterales (*n = *5457) and *P. aeruginosa* (*n = *1949) from 10 medical centres in three African countries (4 medical centres in South Africa, 3 in Morocco and 3 in Nigeria), and 10 medical centres in five Middle East countries (4 medical centres in Israel, 3 in Kuwait, 1 in Jordan, 1 in Qatar and 1 in Saudi Arabia) from 2018 to 2020. Isolates were collected from bloodstream, intraabdominal, respiratory tract, skin and soft tissue, and urinary tract infection specimen sources. Table S1 (available as Supplementary data at *JAC-AMR* Online) cross-references the isolates by year and country of collection and Table S2 stratifies the isolates by year of collection, patient age and patient location. All isolates were transported from individual medical centres to IHMA (Schaumburg, IL, USA) and re-identified by MALDI-TOF mass spectrometry (Bruker Daltonics, Billerica, MA, USA) prior to antimicrobial susceptibility testing. The identities of the species of Gram-negative isolates tested are listed in Table S3. The CLSI broth microdilution method was used to determine MICs that were interpreted using 2021 CLSI breakpoints.^18,19^

### Screening for β-lactamase genes

For Enterobacterales, all isolates with a meropenem MIC >1 mg/L (meropenem intermediate, meropenem resistant), a ceftazidime/avibactam MIC >8 mg/L (ceftazidime/avibactam resistant), or an aztreonam/avibactam MIC >8 mg/L (data not shown) were screened for the presence of β-lactamase genes. In addition, a subset of isolates of *Escherichia coli*, *K. pneumoniae*, *Klebsiella oxytoca* and *Proteus mirabilis* with a ceftazidime or an aztreonam MIC >1 mg/L were also screened for the presence of β-lactamase genes (100% of qualifying isolates from 2018 and 2019 and 50% of qualifying isolates from 2020 were tested). For *P. aeruginosa*, all isolates from 2018 and 2019, and ∼25% of isolates from 2020 with a meropenem MIC >2 mg/L (meropenem intermediate, meropenem resistant) were tested for the presence of β-lactamase genes. Isolates of Enterobacterales and *P. aeruginosa* were screened for the presence of genes encoding ESBLs (SHV, TEM, CTX-M, VEB, PER and GES [e.g. GES-1, GES-7, GES-9, GES-11, GES-12, GES-19]), acquired AmpC β-lactamases (ACC, ACT, CMY, DHA, FOX, MIR, MOX), PDC (the intrinsic, chromosomal AmpC of *P. aeruginosa*), serine carbapenemases (KPC, GES [e.g. GES-2, GES-5, GES-6, GES-15, GES-20] and OXA-48-like [Enterobacterales] or OXA-24-like [*P. aeruginosa*]) and MBLs (NDM, IMP, VIM, SPM, GIM) using published multiplex PCR assays and sequencing for genes encoding β-lactamases as previously described.^14,20^ All detected genes encoding carbapenemases, PDC and ESBLs were amplified using gene-flanking primers and sequenced (Sanger method).

### Ethical approval

Ethical approval was not required.

## Results

Isolates of Enterobacterales were highly susceptible to amikacin (96.7% susceptible), ceftazidime/avibactam (96.6%), and tigecycline (96.0%), and slightly less susceptible to meropenem (94.3%) (Table 1). Isolates of *P. aeruginosa* were most susceptible to ceftazidime/avibactam (89.1%) and amikacin (88.9%); only 73.1% of isolates were meropenem susceptible. The addition of avibactam to ceftazidime increased susceptibility to ceftazidime by 32.4% for Enterobacterales and by 13.2% for *P. aeruginosa*. A total of 3.1% (170/5457) of Enterobacterales isolates and 10.0% (195/1949) of *P. aeruginosa* isolates were both meropenem non-susceptible and ceftazidime/avibactam resistant. In total, 45.7% (143/313) of meropenem-non-susceptible Enterobacterales and 62.8% (329/524) of meropenem-non-susceptible *P. aeruginosa* were susceptible to ceftazidime/avibactam (Table S4).

**Table 1.** *In vitro* susceptibility of isolates of Enterobacterales and *P. aeruginosa* from hospitalized patients in Africa and the Middle East in 2018–20 to a panel of 11 antimicrobial agents with MICs interpreted by CLSI breakpoints

Organism (*n*)/Antimicrobial agent	MIC (mg/L)			MIC interpretation (%)		
	MIC_50_	MIC_90_	MIC range	susceptible	intermediate	resistant
Enterobacterales (5457)						
Amikacin	2	8	≤0.25 to >64	96.7	1.0	2.4
Aztreonam	0.12	64	≤0.015 to >128	64.8	2.5	32.7
Cefepime	≤0.12	>32	≤0.12 to >32	65.1	8.2^a^	26.7
Ceftazidime	0.5	64	≤0.015 to >128	64.2	3.6	32.2
Ceftazidime/avibactam	0.12	0.5	≤0.015 to >128	96.6	NA	3.4
Colistin	0.5	>8	≤0.06 to >8	NA	81.6	18.4
Imipenem	0.25	2	≤0.06 to >8	82.9	8.1	8.9
Levofloxacin	0.5	>8	≤0.25 to >8	61.1	7.9	31.0
Meropenem	≤0.06	0.12	≤0.06 to >16	94.3	0.7	5.0
Piperacillin/tazobactam	2	>64	≤0.12 to >64	83.2	5.4	11.4
Tigecycline	0.5	2	≤0.015 to >8	96.0	3.6	0.4
*P. aeruginosa* ^b^ (1949)						
Amikacin	4	32	≤0.25 to >64	88.9	1.8	9.3
Aztreonam	8	32	≤0.015 to >128	67.9	13.8	18.3
Cefepime	4	32	≤0.12 to >32	77.4	7.2	15.4
Ceftazidime	4	64	≤0.03 to >128	75.9	4.0	20.2
Ceftazidime/avibactam	2	16	≤0.03 to >128	89.1	NA	10.9
Colistin	1	2	≤0.12 to >8	NA	99.6	0.4
Imipenem	2	>8	≤0.06 to >8	63.3	9.5	27.2
Levofloxacin	0.5	>8	≤0.25 to >8	67.2	7.8	25.0
Meropenem	0.5	16	≤0.06 to >16	73.1	6.8	20.1
Piperacillin/tazobactam	8	>64	≤0.12 to >64	72.1	12.6	15.3

NA, not applicable.

a The percentage susceptible-dose dependent (SDD) value is given in the percentage intermediate box for cefepime tested against Enterobacterales; CLSI does not published an intermediate MIC breakpoint for cefepime tested against Enterobacterales.

b Tigecycline is inactive against *P. aeruginosa* and was not tested.

Table 2 provides an overview of the composition of carbapenemase-positive Enterobacterales isolates identified in the study. In total, 1507 isolates of Enterobacterales were tested for β-lactamase content based on screening criteria and 337 isolates (6.2% of the 5457 Enterobacterales isolates tested in the study) carried one or more carbapenemase genes. A total of 188 isolates carried a serine carbapenemase (178 OXA, 10 KPC) and 167 isolates carried an MBL. One hundred and sixty isolates carried an OXA carbapenemase as their only carbapenemase and 18 isolates carried both an MBL and an OXA carbapenemase gene. Only 10 isolates carried a KPC. A linear trend in the percentage of carbapenemase-positive isolates year-to-year (2018 [4.9%, 83/1677]; 2019 [7.5%, 135/1798]; 2020 [6.0%, 119/1982]) was not observed. Serine carbapenemase and/or MBL genes were detected in 89.1% (279/313) of meropenem-non-susceptible isolates of Enterobacterales; MBL genes accounted for 51.8% (162/313) of meropenem-non-susceptible isolates. An MBL gene was identified in 89.1% (163/183) of ceftazidime/avibactam-resistant isolates of Enterobacterales.

**Table 2.** Overview of the composition of carbapenemase-positive Enterobacterales isolates from Africa and the Middle East (combined) and the *in vitro* susceptibility of those isolates to ceftazidime/avibactam

Carbapenemase(s)/β-lactamase(s) identified^a^,^b^,^c^,^d^	*n*	MIC (mg/L)^e^			MIC interpretation (% susceptible)
		MIC_50_	MIC_90_	MIC range	
KPC + AmpC + ESBL + OSBL	1	—	—	1	100
KPC + ESBL + OSBL	3	—	—	0.25 to 0.5	100
KPC + OSBL	6	—	—	0.5 to 4	100
Total	10	1	4	0.25 to 4	100
MBL	11	>128	>128	32 to >128	0
MBL + AmpC	3	—	—	>128	0
MBL + AmpC + ESBL	1	—	—	>128	0
MBL + AmpC + ESBL + OSBL	19	>128	>128	32 to >128	0
MBL + AmpC + OSBL	5	—	—	>128	0
MBL + ESBL	7	—	—	32 to >128	0
MBL + ESBL + OSBL	93	>128	>128	0.06 to >128	4.3
MBL + OSBL	10	>128	>128	>128	0
MBL + OXA	2	—	—	>128	0
MBL + OXA + AmpC + ESBL + OSBL	6	—	—	>128	0
MBL + OXA + AmpC + OSBL	1	—	—	>128	0
MBL + OXA + ESBL + OSBL	8	—	—	>128	0
MBL + OXA + OSBL	1	—	—	>128	0
Total	167	>128	>128	0.06 to >128	2.4
OXA	4	—	—	0.12 to 0.25	100
OXA + AmpC	1	—	—	1	100
OXA + AmpC + ESBL + OSBL	6	—	—	0.25 to 2	100
OXA + AmpC + OSBL	1	—	—	2	100
OXA + ESBL	6	—	—	0.12 to 32	83.3
OXA + ESBL + OSBL	122	0.5	1	0.06 to 4	100
OXA + OSBL	20	0.5	2	0.25 to >128	95.0
Total	160	0.5	1	0.25 to >128	98.8

AmpC, acquired Ambler class C cephalosporinase; OSBL, original spectrum β-lactamase; OXA, Ambler class D OXA carbapenemase.

a The 10 KPCs identified were KPC-2 (7 isolates) and KPC-3 (3).

b The 42 AmpC enzymes (12.5% of isolates tested) identified in the 337 carbapenemase-positive isolates were CMY-4 (21 isolates), CMY-2 (12), CMY-42 (3), CMY-6 (2), CMY-16 (2) and CMY-168 (2).

c The 283 ESBLs identified in 272 (80.7%) of the 337 carbapenemase-positive isolates were CTX-M-15 (227 isolates), SHV-ESBL (23), CTX-M-9-type (13), SHV-12 (7), CTX-M-1-type (4); CTX-M-100 (3), CTX-M-27 (2), CTX-M-65 (2), CTX-M-2 (1), CTX-M-14 (1), CTX-M-32 (1), PER-12 (1), VEB-5 (1) and VEB-9 (1).

d The 438 OSBLs identified in 302 (89.6%) of the 337 carbapenemase-positive isolates were TEM-OSBL (229 isolates) and SHV-OSBL (209).

e MIC_50_ and MIC_90_ values are only shown for isolate counts of ≥10 isolates.

Table 3 shows the identities and distribution of carbapenemases in Enterobacterales for countries in Africa and the Middle East. NDM-1 was the most common MBL identified, accounting for 64.1% (100/156) of NDM enzymes and 59.9% (100/167) of all MBLs detected. MBLs were more frequently identified among isolates from African countries than Middle Eastern countries. NDM-1 was the commonest NDM type in African countries while NDM-5 was the commonest in Middle Eastern countries (primarily due to isolates from Kuwait). All five VIM-positive isolates from South Africa were VIM-1 and all six VIM-positive isolates from both Israel and Kuwait were VIM-4. Of the 10 KPC-positive isolates, 7 were KPC-2 and 3 (all from Israel) were KPC-3. No MBLs were identified in isolates from Jordan; MBLs were present in isolates from all seven other countries. OXA-48 (47.8%, 85/178) and OXA-181 (38.8%, 69/178) were the most commonly detected OXAs overall. OXA-48 and OXA-181 were also more commonly detected in isolates from African countries while OXA-232 and KPC were more frequently identified in isolates from Middle East countries. Qatar had the highest percentage of isolates with a carbapenemase (18.0% of all isolates of Enterobacterales tested from that country) compared with the other seven countries, whereas isolates from Israel had the lowest percentage of carbapenemases (0.9% of all isolates of Enterobacterales tested from that country).

**Table 3.** Distribution of carbapenemases detected in Enterobacterales from Africa and the Middle East stratified by country

Region/Country	Isolates with carbapenemase(s),^a^*n* [% of all isolates tested]/Isolates tested for presence of β-lactamases, *n*/Total isolates tested in study, *n*	MBL^b^								Serine carbapenemase			
		NDM-1	NDM-4	NDM-5	NDM-7	NDM-38	NDM-type	VIM-1	VIM-4	OXA-48	OXA-181	OXA-232	KPC
Africa													
Morocco	72 [8.8]/210/816	32			8					38		1	
Nigeria	69 [7.6]/374/912	42	2	9	8	1				5	5		
South Africa	90 [7.4]/245/1223	14			1			5		13	56	4	2
Total	231 [7.8]/829/2951	88	2	9	17	1	0	5	0	56	61	5	2
Middle East													
Israel	10 [0.9]/233/1098	1							3	1			5
Jordan	7 [5.5]/44/127									5			2
Kuwait	32 [3.8]/243/832	5		13			1		3	7	2	1	1
Qatar	34 [18.0]/75/189	4		5	5					9	6	6	
Saudi Arabia	23 [8.8]/83/260	2		2	1					7		12	
Total	106 [4.2]/678/2506	12	0	20	6	0	1	0	6	29	8	19	8
Grand total	337 [6.2]/1507/5457	100	2	29	23	1	1	5	6	85	69	24	10

MBL-positive isolates per country: Morocco, 40; Nigeria, 62; South Africa, 20; Israel, 4; Jordan, 0; Kuwait 22; Qatar, 14; Saudi Arabia, 5.

Serine carbapenemase-positive isolates per country: Morocco, 39; Nigeria, 10; South Africa, 75; Israel, 5; Jordan, 7; Kuwait 11; Qatar, 21; Saudi Arabia, 19.

a Some isolates carried more than one carbapenemase.

b IMP-positive isolates were not identified in the isolates of Enterobacterales screened.

The most common species of Enterobacterales carrying NDM were *K. pneumoniae* (62.8% of isolates; 98/156), *E. coli* (9.6%, 15/156), *Providencia* spp. (9.0%; 14/156) and *Enterobacter* spp. (7.7%; 12/156). The 11 VIM-positive isolates were *Enterobacter cloacae* (5 isolates) and *E. coli* and *K. pneumoniae* (3 isolates each). The 178 OXA-positive isolates comprised primarily *K. pneumoniae* (78.7%, 140/178), *Enterobacter* spp. (10.1%, 18/178) and *E. coli* (6.7%, 12/178). The KPC-positive isolates included eight isolates of *K. pneumoniae* and two isolates of *E. cloacae*. A total of 12.5% (42/337) of carbapenemase-positive Enterobacterales also carried a plasmid-mediated AmpC enzyme, most commonly CMY-4 (21 isolates) and CMY-2 (12 isolates), and 80.7% (272/337) of carbapenemase-positive Enterobacterales also carried one or more ESBLs, most frequently CTX-M-15 (227 of carbapenemase-positive Enterobacterales isolates; 83.5% (227/272) of ESBL-positive isolates identified). All KPC-positive (100%) Enterobacterales and 98.8% (158/160) of Enterobacterales carrying only an OXA carbapenemase (no MBL) were ceftazidime/avibactam susceptible.

Table 4 provides an overview of the composition of carbapenemase-positive *P. aeruginosa* isolates identified. In total, 364 isolates were tested for the presence of carbapenemase/β-lactamase genes. Of those, 248 isolates (68.1%) were devoid of any β-lactamase and 88 (24.2%) carried one or more carbapenemase genes (4.5% of the 1949 *P. aeruginosa* isolates tested in the study). Eighty-one isolates carried an MBL and eight carried a GES carbapenemase (one isolate carried both a MBL and a GES carbapenemase). Six of the seven (85.7%) GES-positive isolates (no MBL present) were susceptible to ceftazidime/avibactam. A wide variation in susceptibility to ceftazidime/avibactam was observed (0%–100% susceptible) among the subsets of meropenem-non-susceptible, carbapenemase-negative isolates suggesting the presence of alternate resistance mechanisms in some isolates. MBL and/or GES carbapenemase genes were detected in 24.2% (88/364) of the meropenem-non-susceptible isolates of *P. aeruginosa* tested. An MBL gene was identified in 63.5% (80/126) of ceftazidime/avibactam-resistant isolates of *P. aeruginosa* tested.

**Table 4.** Overview of carbapenemase-positive and carbapenemase-negative isolates of *P. aeruginosa* with meropenem-non-susceptible phenotypes from Africa and the Middle East (combined) and the *in vitro* susceptibility of those isolates to ceftazidime/avibactam

Group/Carbapenemase or β-lactamase identified	*n*	MIC (mg/L)^a^			MIC interpretation (% susceptible)
		MIC_50_	MIC_90_	MIC range	
Meropenem non-susceptible, carbapenemase positive					
MBL	65	64	>128	4 to >128	1.5
MBL + ESBL	12	64	128	16 to 128	0
MBL + GES carbapenemase	1	—	—	32	0
MBL + GES ESBL-like	3	—	—	32 to >128	0
GES carbapenemase	5	—	—	2 to 4	100
GES carbapenemase + GES ESBL-like	2	—	—	4 to 32	50.0
Meropenem non-susceptible, carbapenemase negative					
β-lactamase negative	248	4	16	0.5 to 128	89.9
OSBL^b^	1	—	—	8	100
ESBL positive	13	64	128	16 to >128	0
GES ESBL-like	7	—	—	4 to 16	57.1
GES, spectrum undefined	7	—	—	8 to 128	42.9
Grand total	364	8	128	0.5 to >128	65.4

a MIC_50_ and MIC_90_ values are only shown for isolate counts of ≥10 isolates.

b TEM-1.

Table 5 displays the identities and distribution of carbapenemases in isolates of *P. aeruginosa* from countries in Africa and the Middle East. VIM-2 was the most common MBL identified, accounting for 89.8% (53/59) of VIM enzymes and 65.4% (53/81) of all MBLs detected. MBLs were more frequently identified among isolates from countries in Africa than the Middle East. NDM-1 was also detected in isolates from African countries, primarily Nigeria.

**Table 5.** Distribution of carbapenemases detected in *P. aeruginosa* from Africa and the Middle East stratified by country

Region/Country	Isolates with carbapenemase(s), *n* [% of all isolates tested]/Isolates tested for presence of β-lactamases, *n*/Total isolates tested in study, *n*	MBL							Serine carbapenemase		
		NDM-1^a^	IMP	VIM-1	VIM-2	VIM-4	VIM-5	VIM-44	GES-2	GES-5	GES-20
Africa											
Morocco	17 [5.5]/50/311	2			14						1
Nigeria	28 [12.8]/41/218	17			5		3	1	1	1	1
South Africa	18 [4.1]/76/444				16				2		
Total	63 [6.5]/167/973	19	0	0	35	0	3	1	3	1	2
Middle East											
Israel	1 [0.2]/53/420				1						
Jordan	1 [3.0]/8/33					1					
Kuwait	16 [4.7]/89/340			1	13					1	1
Qatar	2 [2.6]/20/77	1			1						
Saudi Arabia	5 [4.7]/27/106		2^b^		3						
Total	25 [2.6]/197/976	1	2	1	18	1	0	0	0	1	1
Grand total	88 [4.5]/364/1949	20	2	1	53	1	3	1	3	2	3

MBL-positive isolates per country: Morocco, 16; Nigeria, 26; South Africa, 16; Israel, 1; Jordan, 1; Kuwait, 14; Qatar, 2; Saudi Arabia, 5.

Serine carbapenemase-positive isolates per country: Morocco, 1; Nigeria, 3; South Africa, 2; Israel, 0; Jordan, 0; Kuwait, 2; Qatar, 0; Saudi Arabia, 0.

a Only NDM-1 was identified in *P. aeruginosa*, no other NDM types were detected.

b The two IMP-positive isolates comprised one isolate with IMP-1 and one isolate with IMP-7.

For patients aged 0–17 years and 18–110 years, notable differences in the percentages of isolates carrying carbapenemases (or the specific type[s] of carbapenemase carried [data not shown]), were not observed for Enterobacterales (0–17 years, 6.4%, 60/936; 18–110 years, 6.4%, 265/4153) or *P. aeruginosa* (0–17 years, 4.3%, 10/235; 18–110 years, 4.9%, 77/1570). For Enterobacterales, carbapenemase positivity was approximately twice as common among isolates from ICU patients (9.6%, 123/1275) than isolates from non-ICU inpatients (5.2%, 175/3381) and emergency room/other patients (4.9%, 39/801); however, trends in the specific type of carbapenemase carried in isolates from ICU versus non-ICU patients were not observed (data not shown). In comparison, for *P. aeruginosa*, rates of carbapenemase positivity were similar among isolates from ICU patients (4.2%, 24/572), non-ICU inpatients (4.5%, 53/1173) and emergency room/other patients (5.4%, 11/204); again, trends in the specific type of carbapenemase carried in isolates from ICU versus non-ICU patients were not observed (data not shown).

## Discussion

In the current study, 96.6% and 94.3% of Enterobacterales isolates were susceptible to ceftazidime/avibactam and meropenem, respectively (Table 1). These results align closely with the identification of 337 of 5457 (6.2%) of Enterobacterales isolates carrying one or more carbapenemase genes; 3.4% of isolates carried a serine carbapenemase (OXA or KPC) and 3.1% carried an MBL (NDM or VIM). Serine carbapenemase and/or MBL genes were detected in 89.1% of meropenem-non-susceptible isolates of Enterobacterales and an MBL gene was identified in 89.1% of ceftazidime/avibactam-resistant isolates. The ceftazidime/avibactam susceptibility results in the current study are consistent with previous reports that avibactam inhibits Ambler class A β-lactamases (e.g. ESBLs, KPCs), class C (AmpC) β-lactamases and certain class D (OXA-48) β-lactamases. Class B, MBLs, are not inhibited by clinically available β-lactamase inhibitors.^12–14,21^ Earlier publications have also shown that ceftazidime/avibactam inhibits carbapenem-resistant isolates of Enterobacterales that express an ESBL and/or overexpress AmpC in combination with porin changes and/or upregulated efflux.^12,13^ The observation that carbapenemases were present in most carbapenem-non-susceptible (serine carbapenemase or MBL) and ceftazidime/avibactam-resistant (MBL) isolates of Enterobacterales from the Africa/Middle East region confirms results published in previous studies describing isolates from this region and confirms that the rates of carbapenemase carriage are substantially higher in the Africa/Middle East region than in some other global regions where non-carbapenemase-mediated mechanisms predominate in carbapenem-non-susceptible Enterobacterales.^3,5,11–13^

In the current study, 89.1% and 73.1% of *P. aeruginosa* isolates were susceptible to ceftazidime/avibactam and meropenem, respectively (Table 1). The majority (68.1%) of carbapenem-non-susceptible isolates tested for the presence of carbapenemase/β-lactamase genes were negative (Table 4), confirming results published in similar studies performed using isolates from other regions.^3,5,11^ Only 24.2% of carbapenem-non-susceptible *P. aeruginosa* carried one or more carbapenemase genes, however, this still represented 4.5% of all 1949 *P. aeruginosa* isolates tested in the study. Most (92.0%, 81/88) carbapenemase-positive *P. aeruginosa* carried an MBL against which ceftazidime/avibactam was inactive. An MBL gene was identified in 63.5% (80/126) of ceftazidime/avibactam-resistant isolates of *P. aeruginosa* tested. Six of the seven (85.7%) GES carbapenemase-positive isolates (no MBL present) were susceptible to ceftazidime/avibactam. A wide variation in susceptibility to ceftazidime/avibactam was observed among the subsets of meropenem-non-susceptible, carbapenemase-negative isolates suggesting the presence of alternate, novel resistance mechanisms in some isolates as ceftazidime/avibactam has been demonstrated to be active against *P. aeruginosa* that are carbapenem resistant due to elevated production of PDC (intrinsic AmpC) in combination with upregulated efflux or porin loss.^14^

In the current study, for Enterobacterales, carbapenemase positivity was approximately twice as common among isolates from ICU patients (9.6%) than isolates from non-ICU inpatients (5.2%) and emergency room/other patients (4.9%); in comparison, *P. aeruginosa* isolates from ICU patients (4.2%), non-ICU inpatients (4.5%) and emergency room/other patients (5.4%) had similar rates of positivity. Trends in the specific type of carbapenemase carried in isolates from ICU versus non-ICU patients were not observed. Previously we had reported that for Enterobacterales in African and Middle Eastern countries both MDR phenotypes and difficult-to-treat resistance (DTR) phenotypes were significantly more common for ICU than non-ICU isolates while for *P. aeruginosa* significant differences in the percentage of MDR or DTR phenotypes among ICU and non-ICU isolates were not observed.^16^

Geographic differences in carbapenemase distribution have been previously described and align with observations made in the current study for the Africa/Middle East region (Tables 3 and 5). KPCs are the primary carbapenemase in Enterobacterales in North America and Latin America; they are also common in Europe together with OXA-48-like carbapenemases.^3,5^ Previous studies have reported that KPC was uncommon in carbapenem-resistant Enterobacterales from the Middle East, except for Israel,^5,22,23^ similar to findings in the current study. MBLs have been reported to be more common than KPCs in the Asia-Pacific (NDM plus IMP) and Africa/Middle East (NDM not IMP) regions.^3,5^ The 2012–17 ATLAS study reported only 2.1% of Enterobacterales isolates from the Africa/Middle East region were carbapenem non-susceptible (1.8% meropenem resistant plus 0.3% meropenem intermediate)^3^ compared with the current study, which found 5.7% carbapenem-non-susceptible Enterobacterales. This difference may reflect the inclusion of isolates from several different countries in the current study (Morocco, Jordan, Qatar and Saudi Arabia) and the exclusion of isolates from Kenya. The 2012–17 ATLAS study also reported 44% of meropenem-non-susceptible isolates carried MBLs (primarily [81%] NDM-type), 27% had an OXA-48-like, 19% a KPC and 6% a VIM.^3^ Other studies have also reported that carbapenem-resistant Enterobacterales in Africa and the Middle East commonly carry NDM and OXA-48-like carbapenemases and rarely KPCs.^5,23–25^

The current study also confirmed the results of earlier global surveillance programmes that reported that carbapenemases are absent in the majority of carbapenem-non-susceptible isolates of *P. aeruginosa*^4,14^ and that when a carbapenemase is present, it is most often an MBL (VIM > IMP > NDM) and not a serine carbapenemase (GES). VIM is the most common carbapenemase in the Africa/Middle East region (Table 5) while NDM and IMP predominate in the Asia/Pacific region.^5,7,14^ In Europe, the Asia-Pacific region and Latin America the greatest percentages of MBL-positive isolates have been identified in Russia, Greece, the Philippines and Venezuela.^5,7,14^ Although the global percentage of *P. aeruginosa* isolates carrying MBLs remains relatively low, they are present in all global regions and the potential for spread clearly exists.

Despite new therapeutic options and promising alternatives currently in different phases of development, increases in MDR infections associated with high morbidity and mortality has continued. There is an urgent need for strong national surveillance data, antimicrobial stewardship programmes and point-of-care diagnostics to identify MDR infections. Implementation of antimicrobial stewardship programmes is key to supporting clinical decisions for better patient outcomes.

Although this multicentre and multiyear study generated a significant amount of standardized antimicrobial susceptibility and molecular data, it has some limitations. The ATLAS global surveillance programme is not intended to evaluate the geographic prevalence of resistance or resistance genes as it annually requests each participating medical centre laboratory collect defined quotas of isolates of selected clinically relevant bacterial species from patients (one isolate/patient/year regardless of hospital location) with specific infection types. The data generated from participating laboratories within a country cannot be extrapolated to represent all isolates or geographic areas within that country as countries had only one to four participating ATLAS sites and the numbers of isolates collected were often low. ATLAS data may over- or underrepresent true rates of antimicrobial susceptibility and resistance gene presence in a country. Therefore, implementation of national surveillance programmes together with better diagnostic techniques in this geographical region can provide complementary data. Finally, not all carbapenem-non-susceptible *P. aeruginosa* isolates were tested for the presence of β-lactamase genes due to financial constraints.

In conclusion, ceftazidime/avibactam was highly active against clinical isolates of Enterobacterales (96.6% susceptible) and *P. aeruginosa* (89.1%) from countries in Africa and the Middle East. However, it is concerning that carbapenemases were identified in 6.2% of all Enterobacterales and 4.5% of all *P. aeruginosa* tested in this study of 2018–20 isolates and that approximately 50% and 92% of carbapenemases in Enterobacterales and in *P. aeruginosa*, respectively, were MBLs. Ceftazidime/avibactam may be a valuable treatment option for infections caused by carbapenem-resistant Gram-negative bacilli that do not carry MBLs, particularly Enterobacterales. Increases in Gram-negative infections caused by isolates carrying carbapenemases, particularly MBLs, will increase the challenge of treating these infections where currently ceftazidime/avibactam in combination with other antibacterial agents may be a viable treatment option. Regional and country-specific differences in carbapenem resistance mechanisms are important when evaluating patient treatment options. Continued reference method antimicrobial susceptibility testing of clinical isolates of Gram-negative bacilli and monitoring for carbapenemases is imperative as geographic variation in carbapenem resistance and carbapenemases was observed for Africa and Middle East countries.

## Supplementary Material

dlac060_Supplementary_Data

## Supplementary data

Tables S1 to S4 are available as Supplementary data at *JAC-AMR* Online.
