# Supplementary material for: Carbapenem-resistant Enterobacterales and Pseudomonas aeruginosa causing infection in Africa and the Middle East: a surveillance study from the ATLAS programme (2018–20)
Source: JAC Antimicrob Resist. 2022 Jun 17;4(3):dlac060. doi: 10.1093/jacamr/dlac060 (PMC9204471; doi:10.1093/jacamr/dlac060)
Supplement: dlac060_Supplementary_Data [file dlac060_supplementary_data.docx]

### **Supplementary data**

### **Table S1.** Numbers of isolates of Enterobacterales and *P. aeruginosa* from Africa and the Middle East collected by the ATLAS Surveillance Program in 2018-2020 stratified by country and year of collection

| Organism | Region | Country | Year of collection  No. of isolates | | | Total number of isolates |
| --- | --- | --- | --- | --- | --- | --- |
|  |  |  | 2018 | 2019 | 2020 |  |
| Enterobacterales | Africa | Morocco | 268 | 270 | 278 | 816 |
|  |  | Nigeria | 233 | 311 | 368 | 912 |
|  |  | South Africa | 364 | 387 | 472 | 1223 |
|  |  | Total | 865 | 968 | 1118 | 2951 |
|  |  |  |  |  |  |  |
|  | Middle East | Israel | 376 | 371 | 351 | 1098 |
|  |  | Jordan | 75 | --^a^ | 52 | 127 |
|  |  | Kuwait | 275 | 281 | 276 | 832 |
|  |  | Qatar | -- ^a^ | 92 | 97 | 189 |
|  |  | Saudi Arabia | 86 | 86 | 88 | 260 |
|  |  | Total | 812 | 830 | 864 | 2506 |
|  |  |  |  |  |  |  |
|  |  | Grand Total | 1677 | 1798 | 1982 | 5457 |
|  |  |  |  |  |  |  |
| *P. aeruginosa* | Africa | Morocco | 79 | 110 | 122 | 311 |
|  |  | Nigeria | 38 | 79 | 101 | 218 |
|  |  | South Africa | 98 | 146 | 200 | 444 |
|  |  | Total | 215 | 335 | 423 | 973 |
|  |  |  |  |  |  |  |
|  | Middle East | Israel | 100 | 157 | 163 | 420 |
|  |  | Jordan | 24 | -- ^a^ | 9 | 33 |
|  |  | Kuwait | 76 | 135 | 129 | 340 |
|  |  | Qatar | -- ^a^ | 38 | 39 | 77 |
|  |  | Saudi Arabia | 26 | 39 | 41 | 106 |
|  |  | Total | 226 | 369 | 381 | 976 |
|  |  |  |  |  |  |  |
|  |  | Grand Total | 441 | 704 | 804 | 1949 |

^a^ Isolates were not collected from laboratories in that country in that year.

### **Table S2.** Numbers of isolates of Enterobacterales and *P. aeruginosa* from Africa and the Middle East (combined) collected by the ATLAS Surveillance Program in 2018-2020 stratified by patient age, patient location, and year of collection

|  |  | Year of collection  Number of isolates | | |  |
| --- | --- | --- | --- | --- | --- |
| Organism | Demographic parameter | 2018 | 2019 | 2020 | Total number of isolates |
| Enterobacterales | Patient age |  |  |  |  |
|  | Pediatric: 0-17 years | 313 | 284 | 339 | 936 |
|  | Adult: 18-103 years | 1247 | 1397 | 1509 | 4153 |
|  | Age not provided | 117 | 117 | 134 | 368 |
|  | Total | 1677 | 1798 | 1982 | 5457 |
|  |  |  |  |  |  |
|  | Patient location |  |  |  |  |
|  | ICU | 400 | 394 | 481 | 1275 |
|  | Non-ICU Inpatient | 1029 | 1146 | 1206 | 3381 |
|  | Emergency Room/Other | 248 | 258 | 295 | 801 |
|  | Total | 1677 | 1798 | 1982 | 5457 |
|  |  |  |  |  |  |
| *P. aeruginosa* | Patient age |  |  |  |  |
|  | Pediatric: 0-17 years | 53 | 73 | 109 | 235 |
|  | Adult: 18-110 years | 357 | 583 | 630 | 1570 |
|  | Age not provided | 31 | 48 | 65 | 144 |
|  | Total | 441 | 704 | 804 | 1949 |
|  |  |  |  |  |  |
|  | Patient location |  |  |  |  |
|  | ICU | 127 | 183 | 262 | 572 |
|  | Non-ICU Inpatient | 266 | 435 | 472 | 1173 |
|  | Emergency Room/Other | 48 | 86 | 70 | 204 |
|  | Total | 441 | 704 | 804 | 1949 |

### **Table S3.** Summary of identities of Gram-negative isolates collected by the ATLAS Surveillance Program in 2018-2020 stratified by geographic region

|  |  | Number of isolates (% of isolates by geographic region) | | |
| --- | --- | --- | --- | --- |
| Gram-negative species | Total number of isolates (%) | Africa |  | Middle East |
| *Citrobacter* spp. (all) | 246 (4.5) | 110 (3.7) |  | 136 (5.4) |
| *Citrobacter freundii* | 104 (1.9) | 56 (1.9) |  | 48 (1.9) |
| *Citrobacter koseri* | 114 (2.1) | 38 (1.3) |  | 76 (3.0) |
| *Enterobacter* spp. (all) | 660 (12.1) | 385 (13.0) |  | 275 (11.0) |
| *Enterobacter cloacae* | 437 (8.0) | 244 (8.3) |  | 193 (7.7) |
| *Escherichia coli* | 1675 (30.7) | 942 (31.9) |  | 733 (29.2) |
| *Klebsiella aerogenes* | 131 (2.4) | 40 (1.4) |  | 91 (3.6) |
| *Klebsiella oxytoca* | 110 (2.0) | 52 (1.8) |  | 58 (2.3) |
| *Klebsiella pneumoniae* | 1667 (30.5) | 880 (29.8) |  | 787 (31.4) |
| *Klebsiella variicola* | 46 (0.8) | 34 (1.2) |  | 12 (0.5) |
| *Morganella morganii* | 170 (3.1) | 92 (3.1) |  | 78 (3.1) |
| *Proteus* spp. (all) | 325 (6.0) | 195 (6.6) |  | 130 (5.2) |
| *Proteus mirabilis* | 250 (4.6) | 153 (5.2) |  | 97 (3.9) |
| *Proteus vulgaris* | 49 (0.9) | 25 (0.8) |  | 24 (1.0) |
| *Providencia* spp*.* | 136 (2.5) | 70 (2.4) |  | 66 (2.6) |
| *Serratia marcescens* | 267 (4.9) | 135 (4.6) |  | 132 (5.3) |
| Other spp. | 24 (0.4) | 16 (0.5) |  | 8 (0.3) |
| Enterobacterales (all) | 5457 (100) | 2951 (100) |  | 2506 (100) |
|  |  |  |  |  |
| *Pseudomonas aeruginosa* | 1949 (100) | 973 (100) |  | 976 (100) |

### **Table S4.** *In vitro* susceptibility of isolates of meropenem-susceptible and meropenem-nonsusceptible Enterobacterales and *P. aeruginosa* from Africa and the Middle East (combined) to 11 antimicrobial agents with MICs interpreted by CLSI breakpoints

|  |  | MIC (mg/L) | | |  | MIC interpretation (%) | | |
| --- | --- | --- | --- | --- | --- | --- | --- | --- |
| Organism (*n*) | Antimicrobial agent | MIC_50_ | MIC_90_ | MIC range |  | Susceptible | Intermediate | Resistant |
| Enterobacterales, meropenem-susceptible (5144) | Amikacin | 2 | 8 | ≤0.25 – >64 |  | 98.5 | 0.6 | 0.8 |
|  | Aztreonam | 0.12 | 64 | ≤0.015 – >128 |  | 67.7 | 2.5 | 29.7 |
|  | Cefepime | ≤0.12 | >32 | ≤0.12 – >32 |  | 68.6 | 8.3^a^ | 23.1 |
|  | Ceftazidime | 0.25 | 64 | ≤0.015 – >128 |  | 67.7 | 3.8 | 28.5 |
|  | Ceftazidime-avibactam | 0.12 | 0.5 | ≤0.015 – >128 |  | 99.7 | NA^b^ | 0.3 |
|  | Colistin | 0.5 | >8 | ≤0.06 – >8 |  | NA | 81.5 | 18.5 |
|  | Imipenem | 0.25 | 2 | ≤0.06 – >8 |  | 87.8 | 8.1 | 4.2 |
|  | Levofloxacin | ≤0.25 | >8 | ≤0.25 – >8 |  | 63.8 | 8.0 | 28.1 |
|  | Meropenem | ≤0.06 | 0.12 | ≤0.06 – 1 |  | 100 | 0 | 0 |
|  | Piperacillin Tazobactam | 2 | 32 | ≤0.12 – >64 |  | 88.2 | 5.3 | 6.5 |
|  | Tigecycline | 0.25 | 2 | ≤0.015 – >8 |  | 96.2 | 3.4 | 0.4 |
|  |  |  |  |  |  |  |  |  |
| Enterobacterales, meropenem-nonsusceptible (313) | Amikacin | 8 | >64 | 0.5 – >64 |  | 66.1 | 6.1 | 27.8 |
|  | Aztreonam | >64 | >128 | ≤0.015 – >128 |  | 16.0 | 1.6 | 82.4 |
|  | Cefepime | >32 | >32 | ≤0.12 – >32 |  | 7.0 | 8.0^a^ | 85.0 |
|  | Ceftazidime | >64 | >128 | 0.12 – >128 |  | 6.7 | 0.6 | 92.7 |
|  | Ceftazidime-avibactam | >64 | >128 | 0.06 – >128 |  | 45.7 | NA | 54.3 |
|  | Colistin | 0.5 | >8 | ≤0.06 – >8 |  | NA | 84.0 | 16.0 |
|  | Imipenem | >8 | >8 | 0.25 – >8 |  | 3.8 | 9.3 | 86.9 |
|  | Levofloxacin | >8 | >8 | ≤0.25 – >8 |  | 15.7 | 7.3 | 77.0 |
|  | Meropenem | >16 | >16 | 2 – >16 |  | 0 | 13.1 | 86.9 |
|  | Piperacillin Tazobactam | >64 | >64 | 8 – >64 |  | 1.0 | 7.0 | 92.0 |
|  | Tigecycline | 0.5 | 2 | 0.12 – 8 |  | 92.0 | 7.0 | 1.0 |
|  |  |  |  |  |  |  |  |  |
| *P. aeruginosa*^c^, meropenem-susceptible (1425) | Amikacin | 4 | 8 | ≤0.25 – >64 |  | 97.2 | 1.0 | 1.8 |
|  | Aztreonam | 4 | 16 | ≤0.015 – 128 |  | 80.1 | 10.1 | 9.8 |
|  | Cefepime | 2 | 8 | ≤0.12 – >32 |  | 90.2 | 5.0 | 4.8 |
|  | Ceftazidime | 2 | 16 | ≤0.03 – >128 |  | 87.9 | 2.9 | 9.2 |
|  | Ceftazidime-avibactam | 2 | 4 | ≤0.03 – >64 |  | 98.8 | NA | 1.2 |
|  | Colistin | 1 | 2 | 0.12 – >8 |  | NA | 99.6 | 0.4 |
|  | Imipenem | 2 | 4 | ≤0.06 – >8 |  | 83.9 | 11.6 | 4.5 |
|  | Levofloxacin | 0.5 | 8 | ≤0.25 – >8 |  | 81.4 | 6.7 | 11.9 |
|  | Meropenem | 0.5 | 1 | ≤0.06 – 2 |  | 100 | 0 | 0 |
|  | Piperacillin Tazobactam | 4 | 64 | ≤0.12 – >64 |  | 86.3 | 7.4 | 6.2 |
|  |  |  |  |  |  |  |  |  |
| *P. aeruginosa*^c^, meropenem-nonsusceptible (524) | Amikacin | 8 | >64 | ≤0.25 – >64 |  | 66.2 | 4.0 | 29.8 |
|  | Aztreonam | 16 | >64 | 1 – >128 |  | 34.7 | 23.9 | 41.4 |
|  | Cefepime | 16 | >32 | 0.25 – >32 |  | 42.4 | 13.2 | 44.5 |
|  | Ceftazidime | 16 | 128 | 1 – >128 |  | 43.3 | 6.7 | 50.0 |
|  | Ceftazidime-avibactam | 8 | >64 | 0.5 – >128 |  | 62.8 | NA | 37.2 |
|  | Colistin | 1 | 2 | ≤0.12 – 4 |  | NA | 99.6 | 0.4 |
|  | Imipenem | >8 | >8 | 0.5 – >8 |  | 7.3 | 3.8 | 88.9 |
|  | Levofloxacin | 8 | >8 | ≤0.25 – >8 |  | 28.4 | 10.9 | 60.7 |
|  | Meropenem | 16 | >16 | 4 – >16 |  | 0 | 25.2 | 74.8 |
|  | Piperacillin Tazobactam | 64 | >64 | 2 – >64 |  | 33.4 | 26.7 | 39.9 |

^a^The % susceptible-dose dependent (SDD) value is given in the % intermediate box for cefepime tested against Enterobacterales because the CLSI does not published an intermediate MIC breakpoint for cefepime tested against Enterobacterales.

^b^NA, not applicable.

^c^Tigecycline is inactive against *P. aeruginosa* and was not tested.
